# Competency-Based Health Professions Education in Evolution: Challenges and Adaptations After Implementation

**DOI:** 10.5334/pme.2580

**Published:** 2026-09-29

**Authors:** Robert Englander, Mary Ellen Goldhamer, Aleda M. H. Chen, Anna Oswald, Adelle Atkinson, Holly Caretta-Weyer, Teresa M. Chan, Fremen Chihchen Chou, Timothy R. Dalseg, Reshma G. Kini, Roberta I. Ladenheim, Leila Niemi-Murola, David A. Turner, Linda Snell, Andrew K. Hall, As members of the International Competency-Based Health Professions Educators Collaborative

**Affiliations:** 1 Department of Medical Education, University of Illinois College of Medicine, Chicago, Illinois, USA; 2 Harvard Medical School, Director of Faculty Continuing Medical Education, Division of General Internal Medicine, Massachusetts General Hospital, USA; 3 Harvard Kidney Urology Hematology Training Institute, Boston, Massachusetts, USA; 4 University of Arkansas for Medical Sciences, Little Rock, Arkansas, USA; 5 Faculty of Medicine and Dentistry, University of Alberta, Edmonton, Canada; 6 Royal College of Physicians and Surgeons of Canada in Ottawa, Canada; 7 University of Toronto, Temerty Faculty of Medicine, Canada; 8 Department of Pediatrics and Consultant Immunologist, Division of Immunology and Allergy, The Hospital for Sick Children, Toronto, Canada; 9 Stanford University School of Medicine, Palo Alto, California, USA; 10 Toronto Metropolitan University, Brampton/Toronto, Ontario, Canada; 11 McMaster University, Hamilton, ON, Canada; 12 The Chinese University of Hong Kong Shenzen, Shenzen, Guangdong, China; 13 Institute of Health Policy, Management and Evaluation, University of Toronto, Toronto, ON, Canada; 14 Center for Faculty Development, China Medical University Hospital, Taiwan; 15 School of Medicine, China Medical University, Taichung, Taiwan; 16 University of Toronto, Toronto, Canada; 17 Father Muller Medical College, Mangaluru, India; 18 Universidad Hospital Italiano de Buenos Aires, Buenos Aires, Argentina; 19 University of Helsinki, Finland; 20 Helsinki University Hospital, Helsinki, Finland; 21 Innovation and Competency-Based Education, American Board of Pediatrics, Chapel Hill, North Carolina, USA; 22 McGill University in Montreal, Canada; 23 Royal College of Physicians and Surgeons of Canada, Ottawa, Canada; 24 Department of Emergency Medicine, University of Ottawa, Canada

## Abstract

Health professions education is experiencing a paradigm shift, from a focus on the structures and processes of education to a focus on the outcomes health professionals must demonstrate to meet the healthcare needs of the public served. The Competency-based Health Professions Education (CBHPE) movement now has two decades of implementation experience in some contexts. Educators across health professions working on the design, planning, implementation, or maintenance of competency-based systems can benefit from the lessons learned through implementation efforts to date. In this manuscript, the authors capitalize on the experiences of the International Competency-based Health Professions Educators Collaborative (ICBHPE), a multinational and interprofessional group of educators, to summarize some of the common challenges faced and adaptations made after implementation. The lens of post-implementation challenges allows a view of CBHPE programs as they have matured. The challenges are organized by the five core components of CBHPE: outcome competencies, sequenced progression, tailored learning experiences, competency-focused instruction, and programmatic assessment. For each component, the authors review those challenges that are most common and provide examples of adaptations that have been successful. The challenges and their adaptations are synthesized through “lessons learned” to provide practical insights for designing, planning, implementing, and maintaining CBHPE programs to continue driving the evolution of CBHPE globally.

## Introduction

The paradigm shift in health professions education to a competency-based model began in earnest around the turn of this century [1,2]. Early literature came predominantly from Competency-based Medical Education (CBME) and focused on the theory and implications for implementation [3]. The literature has expanded to include other health professions; thus, Competency-based Health Professions Education (CBHPE) will be the focus throughout this paper. Much has been published about the critical steps educators must take, both before and during initial implementation [4,5,6]. As implementation enters a third decade in some countries and professions, those focused on CBHPE and its implementation can examine the challenges faced and the adaptations needed to enable successful adoption. Implementation science literature clearly highlights the need to engage in the adaptation of both the innovations and their implementation strategies, and to further focus on the subsequent measurement of the impact of these adaptations [7]. At national, regional, institutional, and program levels, competency-based programs are engaging in a broad array of evaluation efforts [8], providing valuable information that is guiding the *evolution* of CBHPE over time.

The International Competency-based Health Professions Educators (ICBHPE) Collaborative is a global group of scholars seeking to catalyze the paradigm shift to CBHPE by fostering a learning community that produces peer-reviewed literature, online blogs, consultations, courses, presentations, and workshops at meetings across the globe. In this paper, we capitalize on the Collaborative’s lived experience across different contexts and professions by exploring examples of CBHPE in evolution. Our aim is to examine the challenges and resultant adaptations that sustain CBHPE after implementation and continuously improve CBHPE program fidelity. The lens of post-implementation challenges allows a view of CBHPE programs as they have matured. In this paper, we organize key examples of CBHPE by its five Core Components: outcome competencies, sequenced progression, tailored learning experiences, competency-focused instruction, and programmatic assessment [9].

This paper is designed to aid those in CBHPE implementation and revision to anticipate the key challenges within each of the core components and to highlight adaptations that have been successful in the field to date. Recognizing the importance of context in program implementation, we present examples from across the globe at both the macro (e.g., national initiatives) and micro levels (e.g., pilot programs). Throughout the text, several competency frameworks are mentioned (such as CanMEDS [10] and the ACGME Core Competencies [11]), as well as frameworks for curriculum and assessment (such as Entrustable Professional Activities [12]). Regardless of the framework, the challenges faced and the adaptations made were similar and generic to competency-based education in the health professions writ large.

## Challenges and Adaptations in Outcomes Competency Frameworks (Core Component #1)

The first core component of a CBHPE program is the clear articulation of outcome competencies [9]. These competencies are generally developed at a macrosystem level, such as a national specialty committee [13] or an entire health profession at a national level [14,15]. Outcome competencies are generally developed by a panel of experts using structured consensus-building methods, such as the nominal group technique or Delphi processes [16].

Two common challenges emerged for this core competency. First, many CBHPE programs experienced that some (or even most) learners could not be deemed competent on all stated outcomes in the first iterations. Often, the program required several iterations of its outcomes competency framework to get its learners to the desired outcomes. CBHPE implementers must therefore develop *a priori* program evaluation strategies that monitor the capacity for learners to reach the desired level of competence as articulated. Second, imported competency frameworks (either from other systems or derived at the macrosystem level) do not fit perfectly in new settings and require either consensus methods to adapt them to local systems- or program-level modifications to allow for effective use. This balance between standardization across sites and systems with local flexibility to build and use systems that work well in specific contexts needs continual consideration. Below, we describe examples of these two common challenges along with adaptations that have been used to address them.

### Challenge #1: Getting learners to the articulated outcomes

A nearly universal challenge has been the inability to have all learners demonstrate the desired level of competence across all the defined outcomes. An example of this is illustrated by the implementation of the Core EPAs for Entering Residency [17] as a component of teaching and assessing competence in undergraduate medical education (UME) at the University of Minnesota in the US. The goal was for at least 90% of students to achieve the “indirect supervision” level of entrustment by the end of their core clerkships. During the first 18 months of implementation, the goal was achieved for only 7 of the 13 defined EPAs. To address this gap, three adaptations were used: 1) altering the EPA description; 2) altering the curriculum and/or opportunities for assessment; and 3) elimination of the EPA as a requirement. An example of an EPA that required altering the description was “Identify system failures and contribute to a culture of safety and improvement.” By limiting the focus of teaching and assessing this EPA to specific functions – identifying system errors, activating the system through event reports, and participating in system-wide safety initiatives – gains were made towards the learner entrustment goal. An EPA that required adapting the curriculum and/or opportunities for assessment was “Give or receive a patient handover to transition care responsibility”. The assessment team recognized that most observations/assessments occurred in the required Intensive Care Unit (ICU) experience of the fourth year and subsequently designated this EPA as the major focus during that rotation. Finally, “Obtain informed consent” was the only EPA for which there was no alteration of the EPA itself or enhancement of the curriculum that allowed learners to reach the desired level of entrustment. Here, relevant procedures were identified as being under the purview of other professionals; thus, the EPA could not be considered a potential graduation requirement.

### Challenge #2: Generic competency outcomes that require local adaptations

A second challenge has arisen when nationally or internationally developed competency frameworks do not fully align with local health systems, professional identities, or clinical practice. Programs may initially implement the framework as written, only to discover that some competencies do not “fit” their context, cannot be assessed meaningfully, or fail to capture what is valued in local practice. As a result, adaptation of the framework is needed to support effective and sustainable implementation. Before examining specific examples, it is critical to recognize *a priori* that the adaptation of competency frameworks from other countries or of a national framework within a country to a specialty ideally occurs at the macro-system level; that is, at the national or specialty level. At this level, the adaptations can still be studied across programs (e.g., undergraduate or graduate programs), allowing benchmarking and comparisons and ensuring all learners across programs at a similar level achieve the desired outcomes (i.e., “comparing apples to apples”). If the adaptations occur at a meso-system level (such as a school or consortium of graduate programs) or at the micro-system level (such as an individual program), these comparisons become impossible (i.e., “comparing apples to oranges”). A clear example of this second challenge comes from the South African Health Professions Council (HPCSA) national adaptation of the Canadian framework, CanMEDS [10], to “AfriMEDS” [18,19]. Early implementation revealed that the central role of *Medical Expert* did not reflect the interprofessional, team-based, and community-oriented nature of South African healthcare. In response, the HPCSA replaced it with the broader role of *Health Practitioner*, explicitly anchoring the framework in local patterns of work and expectations of professional identity [18]. In anesthesiology specifically, a Delphi process identified two domains (*Humaneness* and *Context Awareness)* that clinicians considered essential but were missing from the imported framework [20]. Similar analyses in medicine and dentistry also led to reinterpretation to better align with South Africa’s health priorities and patient population [21,22].

A parallel pattern emerged in Taiwan following the national adoption of the Accreditation Council for Graduate Medical Education (ACGME) Core Competencies. Early implementation showed that several competencies and milestones were difficult to operationalize or assess within the local training environment. In response, specialty committees engaged in a structured process of “glocalization,” revising and reframing competencies to reflect Taiwanese clinical realities [6]. Specific work in family medicine in Taiwan confirmed that some ACGME sub-competencies lacked contextual “fitness,” prompting modification [23].

In India, national mandates for postgraduate CBME articulated extensive competencies but provided limited guidance on how to operationalize them into assessable workplace activities [24]. In one discipline, faculty from multiple institutions across different regions collaboratively developed, refined, and validated a set of national discipline-specific EPAs [25]. This bottom-up adaptation addressed the absence of an actionable national-level assessment and curriculum framework during initial implementation.

Across these settings, the trajectory was similar: a global framework was adopted, early implementation revealed misalignment and challenges, and the framework itself was adapted to fit local needs. These cases highlight that even the most established competency frameworks cannot be assumed to transfer seamlessly across settings. Meaningful implementation often requires reshaping the framework such that it reflects local values, systems, and practices.

## Challenges and Adaptations in Sequenced Progression (Core Component #2)

The second core component of a CBHPE program is sequenced progression. This core component underscores the importance of outlining a sequential pathway to competence that focuses on each next step in development, while affording flexibility as each learner progresses [9]. Two common challenges emerged for this core competency. First, many programs simply skipped this step when developing their competency frameworks. With their competencies delineated, some moved right to the design of the curriculum and program of assessment. The second major challenge to sequenced progression occurred when the curriculum sequencing did not align with the progression of competencies. Below, we describe examples of these two common challenges along with the primary adaptations to address them.

### Challenge #1: Lack of progression markers

In the US in 1999, the ACGME and the American Board of Medical Specialties (ABMS) endorsed six core competency domains, each with delineated competencies within them, as the foundation for physician training and practice [11,26,27]. This “Outcome Project” mandated that all US ACGME-accredited programs use the core competencies as the framework for assessment, curriculum, and certification recommendations. Despite its intent, implementation proved challenging due to a lack of shared understanding by trainees, faculty, and program directors as to how the competencies applied across all specialties or what the expected trajectory of competency acquisition (sequenced progression) in GME training looked like. To address this challenge, the ACGME launched the Milestone Project [28], which created developmental narrative Milestones in each specialty for all of the competencies across the six core competency domains. For the first time, GME program directors had a road map for sequencing the developmental progression of competence in their specialty or sub-specialty. As they were implemented, however, evaluation revealed that because the milestones were specialty-specific, cross-specialty comparisons were difficult. The ACGME is now in its second iteration of the Milestones with standardization across four of the core competencies (Interpersonal and Communication Skills, Professionalism, Systems-based Practice, and Practice-based Learning and Improvement), reserving specialty variation for the Patient Care and Medical Knowledge competencies [29].

### Challenge #2: Learning experiences and developmental outcome expectations are not aligned

Following implementation, many CBHPE programs have identified misalignment between their learning experiences and the intended developmental progression of competence. Several broad concepts underpin developmental progression of competence, including: a) sequencing should progress from care of common and simple problems to more complex ones; b) physician development progresses from dependence on external influences to a more intrinsically driven and regulated process; and c) the development of some competencies involves progressing from knowledge to competence (the ability to demonstrate a skill in context) to capability (the ability to adapt to new contexts) [30].

Tailored learning experiences that conflict with these principles of developmental progression can interfere with learning. An illustration occurred in implementing the competencies for Pediatrics in the US. At the time of implementation, programs were required to have both newborn care and neonatal intensive care (NICU) experiences within the residency program. In many programs, however, residents completed NICU experiences well before their newborn nursery experiences, violating the concept of simple-to-complex above. The resultant simple adaptation was the revision of rotation schedules to ensure newborn nursery care always preceded the NICU experience, thus tailoring the learning experiences to better support the sequenced progression of competence.

## Challenges and Adaptations in Tailored Learning Experiences (Core Component #3)

The third core component of a CBHPE program is the provision of tailored learning experiences that facilitate the development of competence over time. These experiences should include settings that model practice, are flexible to accommodate individual learner needs, and are chosen and sequenced in alignment with established competencies [9]. Two major challenges emerged for this core competency. First, in many programs, the existing curriculum does not provide all learners with the tailored experiences to teach and assess some outcomes. Second, many programs lack the ability to provide graduated autonomy tailored to enable seamless transitions into practice. Below, we describe examples of these two common challenges and adaptations used to address them.

### Challenge #1: Inability to provide/access tailored learning experiences

During CBHPE implementation, many programs have faced the challenge of either lacking adequate experiences to enable learners to attain the desired level of competence or having the experiences in place without learners being aware of them or able to access them. An example of both occurred during the implementation of the Education in Pediatrics Across the Continuum (EPAC) four-site pilot in the United States [31,32]. EPAC was designed to test the feasibility of competency-based time-variable transitions from medical school to residency and from residency to practice or fellowship. For the former transition, students were required to achieve entrustment on the 13 Core EPAs for Entering Residency [17]. For the EPA, “Perform the general procedures of a physician”, students across the pilot schools frequently failed to attain the desired level of entrustment by the end of their core clerkships. Root cause analysis revealed that programs lacked a standardized curriculum for the procedures outlined and could not reliably ensure opportunities for performing them in the clinical setting. The primary adaptations were to expand the list of procedures included in the EPA and to ensure adequate learning and assessment opportunities in the required clerkship rotations.

### Challenge #2: Providing tailored learning experiences for progressive autonomy

There is broad evidence in the medicine and nursing education literatures that some program graduates are unprepared for the transition to fully unsupervised practice, while others achieve competency early and would benefit from increased autonomy [33,34,35,36,37,38]. Fixed-time programs and legal considerations, such as supervision-level requirements for safety and billing purposes, pose strong barriers to graduated autonomy over time. The primary adaptation for this challenge is the engineering of a structured transition that incorporates a period of learner-specific graduated autonomy, usually with an emphasis on preceptorship/mentoring, coaching, and reflection. These transition programs might be incorporated into the pre-graduation program or provided as an onboarding experience after the transition to unsupervised practice.

One example of a transition program within residency (prior to the transition to unsupervised practice) is “Promotion in Place” (PIP), a specific model of competency-based time-variable GME training designed to address this challenge by providing qualifying participants a unique period of “sheltered independence” as fully graduated, credentialed, and certifying Board-eligible physicians until what would have been their regularly scheduled graduation date [39,40]. This model represents an adaptation to time-variable training, allowing practice in a “sheltered” environment with trusted mentors, an experience valued across participant groups [41]. The TIMELESS model from internal medicine at the University of Cincinnati takes a similar approach, allowing qualified residents to progress to distant oversight supervision while not making them the physician of record for billing and legal purposes. This solution addresses the ingrained bureaucracy of the US healthcare system, where multiple regulatory bodies have regulations that curtail what is possible [42]. Both PIP and TIMELESS provide tailored learning experiences with graded autonomy that allow residents the opportunity for time-variable, competency-based advancement [43].

The nursing literature, while not solely focused on competency-based education, is replete with examples of intentionally designed extended (6 month–1 year) programs as part of the on-boarding in a first unsupervised practice experience [44,45]. This literature suggests that key ingredients to successful transition programs include trained preceptors in graduated autonomy, a formal skills curriculum (both clinical and professional), often including simulation, and guided reflection/mentorship. Outcomes include higher competency ratings across domains, increased confidence of the nurses, decreased stress, and improved retention.

## Challenges and Adaptations in Competency-Focused Instruction (Core Component #4)

The fourth core component of a CBHPE program is competency-focused instruction. All instruction, including coaching and feedback, should be designed to help learners develop competence [9]. Program challenges in this core component have been twofold. First, too often, feedback and coaching are not focused on the delineated competencies from core component 1. Second, instruction, feedback, and coaching might be too focused on a specific competency or skill, resulting in missing the “forest” of competency development over time in favor of the “trees” of the one-off feedback session. Below, we describe examples of these two common challenges and the adaptations used to address them.

### Challenge #1: Faculty coaching or feedback not related to the competencies

Transitioning to a CBHPE framework often increases the need for specific competency-focused coaching and/or feedback. This may take time to evolve [46], and faculty can find the evolution to programmatic, competency-focused, workplace-based assessment systems challenging [47]. Sources of confusion for frontline faculty include the introduction of novel feedback forms and rating scales, and expectations for written comments [48] and newly designed, often online, systems [49]. Additional challenges arise from the multiple roles that individuals hold within the CBHPE system (e.g., teacher, assessor, and patient safety monitor). Tensions among these roles can complicate the provision of high-level competency-focused feedback to trainees.

Learner-oriented adaptations have focused on helping trainees actively seek targeted observation and feedback. One such adaptation at the University of Saskatchewan in Canada involved the creation of custom laminated cards that learners could attach to their lanyards during off-service rotations (e.g., rotations where they were not in their primary specialty) to ensure residents knew what EPAs were best taught and assessed in each of their rotations outside of their primary specialty [50], to ensure the residents would receive feedback and coaching designed specifically for the competencies assessed for that rotation. Faculty-oriented adaptations have included several interventions to strengthen coaching and feedback skills, a critical post-implementation need in many CBHPE programs [51,52,53,54]. Other interventions include data systems that provide faculty with useful insights via dashboards [55] or scoring rubrics that assess the quality of faculty comments [56]. Some educators have created automated tools that score comments. These tools harness machine learning and natural language processing to train artificial intelligence (AI) to help faculty understand the gaps in their feedback or coaching language [57].

### Challenge #2: Lack of focus on development: missing the assessment forest for the trees

Competency acquisition requires learners to view their progression in a developmental context over time. Programs implementing CBHPE may provide teaching and assessment that focus on the acquisition of competence *within* a typical course or clinical rotation but may find it more challenging to provide competency-focused instruction longitudinally *across* courses and clinical rotations. Without such integration, learners may receive fragmented feedback that is difficult to synthesize into actionable insights for progression. In response to this challenge, programs have used two primary adaptations: a) implementing a technological platform that organizes longitudinal data in accessible, interpretable formats to optimize learners’ insights and/or b) developing coaching programs that encourage reflection on longitudinal progress over time.

One multi-institutional adaptation that addresses the technological platform to allow longitudinal synthesis of assessment data comes from the Society for Improving Medical Professional Learning (SIMPL) [58,59,60]. SIMPL is used by over 400 training programs in 28 specialties across 6 countries and provides a technological platform with operating room-based and bedside-based modules for assessment and analytics. The platform facilitates assessments in EPAs and Observable Professional Activities (OPAs) [61], providing programs, faculty, learners, and certification boards with actionable longitudinal data.

At the program level, an example of the use of coaching and feedback to ensure longitudinal synthesis of assessment data evolved at the University of Minnesota Medical School during the implementation of an EPA framework for clinical instruction and assessment [62,63]. To stimulate longitudinal reflection and coaching, each student was assigned to an Assessment and Coaching Expert (ACE). These were faculty focused on the EPA teaching and assessment program. Coaches and students together use longitudinal EPA assessment data to develop competency-focused instruction plans for future rotations, improving the students’ use of their data for growth.

## Challenges and Adaptations in Programmatic Assessment (Core Component #5)

The fifth CBHPE core component is programmatic assessment [9]. Central to programmatic assessment is the intentional selection and combination of multiple assessment types and sources. In aggregate, this creates a system of assessment that provides a robust representation of learner competence over time [64,65]. Programmatic assessment has been the most studied of the CBHPE core components. As a result, we identified five common challenges to this core component, including: burden of assessment, feasibility of assessment, technology for assessment, performance-focused assessment, and inadequate assessment tools to allow progression decisions. Program maturation across settings has invariably involved iterative changes in programs of assessment to address these challenges. Therefore, CBHPE programs in any phase-planning, design, implementation, or maintenance-would do well to develop *a priori* measures to determine the success of the assessment program to allow continuous improvement. Specifically, evaluation efforts to uncover the five specific challenges noted above and elaborated through examples below are important to establishing a continuous improvement approach to the program of assessment. Below, we describe examples of these five common challenges to the program of assessment and the adaptations used to address them.

### Challenge #1: Perceived Burden of Assessment

Perhaps the most common challenge is the perceived burden of assessment. For example, through the implementation of Competence by Design (CBD) in Canadian specialist postgraduate medical training, faculty and program leaders reported a high burden of assessment [66,67,68,69]. Program evaluation revealed several contributing factors [67], including: expectations of high volumes of assessment; heavy reliance on workplace based assessments (WBA) over other assessment strategies; misalignment between required contexts for assessments and availability of clinical opportunities in these contexts; challenging technology for assessments; variable institutional and leadership support; and an overarching lack of perceived value of the assessment process by supervisors and learners. Further, program and discipline-specific analyses highlighted that the burden of assessment was highly influenced by the pre-implementation assessment structure. Programs with prior regular daily assessment practices faced much less difficulty than those where assessments were previously infrequent. Key adaptations employed by local programs [70] and published in CBD 2.0 [71] included reframing EPA observation numbers as flexible targets instead of absolute requirements and explicitly positioning them as one piece of a larger program of assessment. Adaptations highlighted the shared responsibility between faculty and learners in the facilitation and timely completion of EPA observations. Individual programs identified local and specialty-specific innovations that would support the opportunities for feedback, coaching, and completion of WBAs. These adaptations were designed to reduce the assessment burden, enhance perceived value, and support sustainable CBHPE programs.

### Challenge #2: Feasibility Challenges in Collecting and/or Using Assessment Data

Feasibility challenges in collecting and using assessment data provide a pressure point in the evolution of programmatic assessment. As programs increase the volume and diversity of workplace-based assessments and place greater emphasis on longitudinal synthesis, traditional processes prove inadequate. Digital systems intended to support CBHPE may be inadequate as they may negatively influence both the capture and organization of data and the evolution of assessment practices [72].

National experiences in Finland and Taiwan illustrate how different governance structures have generated similar adaptations in response to these feasibility pressures. Finland implemented programmatic assessment through a top-down mandate requiring all universities and specialties to adopt competency-based frameworks. In this case, a national electronic assessment system was created that could support structured WBA in a country where such practices had not previously been established [73]. Taiwan, however, followed a bottom-up pathway, beginning with small pilot programs and local innovations, ultimately resulting in the emergence of a prominent model [74] which was adopted by the accreditation body to guide the development of a digital system nationally [75].

Despite these adaptations, both contexts encountered a common tension once national systems were introduced. Platforms designed to address feasibility challenges inevitably introduce uniformity that may conflict with established specialty-specific assessment structures. In both Finland and Taiwan, specialties with longstanding educational models sometimes found national templates restrictive and developed blended approaches to maintain coherence with local practice while meeting national requirements. Interestingly, in this case, established assessment structures resulted in increased challenges to adoption. However, in the burden of assessment challenge outlined earlier, established programs of assessment often made the assessment burden easier to navigate.

### Challenge #3: Data Inadequacy for Progression Decisions

A central premise of programmatic assessment is the use of aggregated data from several sources to provide a full picture of a learner’s competence and developmental trajectory [76]. These data are often designed for use by competency committees to render defensible decisions around learner readiness for progression. However, those who have implemented CBHPE often note challenges in obtaining the data needed to make progression decisions. Challenges range from poor quality or disconfirming data [77] to challenges with data accessibility, visualization, or synthesis [78,79].

One such example can be seen in the Core EPAs for Entering Residency pilot in the US [80]. Four of the ten pilot schools convened a Trained Entrustment Group (TEG) to collate and review all available assessment data and render theoretical summative entrustment decisions for EPAs implemented at their school. They then evaluated the extent to which each school was able to compile sufficient data to make summative entrustment decisions for a given class, examined differences between EPAs on learners’ readiness for distant supervision (the desired level for readiness for residency), and looked at the volume of WBAs needed to make a summative entrustment decision [81]. Each TEG used a different combination and amount of data, including WBAs, end-of-clerkship assessments, objective structured clinical examinations (OSCEs), simulation, and narrative data. TEGs were much more likely to render a decision when WBA data were available. In contrast, the absence of WBA data far more frequently led to the inability to render a decision. Decision confidence was highest for EPAs assessed more frequently and longitudinally across the curriculum. Wide variability existed between and within pilot schools in the number of WBAs needed to make a decision for a given student [81]. These findings prompted adaptations to programmatic assessment. The first adaptation was an increase in the volume of assessment data. A second adaptation was to ensure a variety of data streams to supplement the WBAs, particularly in areas where the overall number of WBAs was lower or longitudinal data was not available [81]. Schools also expanded targeted faculty development to optimize narrative feedback within their programmatic assessment offerings, enhancing the quality of data reviewed by the TEGs in rendering their summative entrustment decisions [82].

### Challenge #4: Performance-focused Assessment Systems

Operationalizing the dual-purpose of assessment *for* learning and assessment *of* learning in CBHPE has been challenging across many settings [65,83]. CBHPE tends to focus on summative assessment rather than building formative assessment programs to drive coaching and learning. The unintended consequences of overly performance-focused assessment systems can be the degradation of feedback [84], assessment being viewed as ‘tick-boxing’ with maladaptive assessment-seeking behavior by trainees [85], and a loss of authenticity in assessment moments [86].

During the initial implementation of postgraduate medicine specialist training in CBD in Canada, evaluation findings suggested that, in several programs, performance-focused assessment was causing unintended consequences such as those above, as well as a negative impact on trainee wellness [66]. In CBD 2.0, several adaptations were put in place to shift the focus from performance- to growth-focused assessment, including: expanding programs of assessment to include an array of tools beyond workplace-based EPA assessments; prioritizing narrative assessments over numerical ratings; allowing space and opportunity for “ultra-low-stakes” [87] assessment with the purposes of feedback and coaching, and optimizing Competence Committee processes to encourage growth-focused coaching for all rather than binary performance-focused progression decisions alone [71].

### Challenge #5: Lack of High-Utility Workplace-based Assessment Tools

WBA is a foundational component of programmatic assessment [88], yet many health professions education programs lack useful WBAs that collect meaningful data to inform robust decision-making. Local assessment tool development in isolation is resource-intensive and may not be driven by best practices in educational design. Further, this can lead to inconsistency across programs and variability in outcomes, constraining discipline-level program evaluation. As an example, in the initial CBHPE implementation, the US pediatric graduate medical education community committed to CBHPE for training and certification, but an authentic national competency-based program of assessment was not employed, resulting in variability in outcomes at the conclusion of training [35,89,90].

To address this issue, the American Board of Surgery began national implementation of a smartphone-based approach to EPA-based WBA in 2023. The surgery community has now accumulated over 500,000 standardized WBAs supporting formative learner development while also addressing curricular gaps locally and nationally, providing data towards national program evaluation, and ultimately improving assurance to the public [91]. Subsequently, the American Board of Pediatrics made an adaptation by partnering to develop a smartphone-based WBA tool based on an EPA framework [92]. This tool was designed to improve WBAs by indexing frontline formative WBAs on the outcomes of interest (the EPAs), creating a direct connection between the core outcomes and programmatic assessment. Large-scale pilot implementation is ongoing, with early unpublished data demonstrating feasibility, improved learner feedback, enhanced development of learning plans, and more meaningful competency committee discussions and decision-making. Similar adaptations are underway in several other US medical specialties to standardize and improve the approach to WBA as an aspect of programmatic assessment in their specialties [93,94,95,96].

## Lessons learned from evolving CBHPE programs

Several lessons emerged from the overview of implementation challenges and adaptations highlighted above. One finding is certain – all implementations of CBHPE experienced challenges. Those engaging in active evaluation and resultant adaptation seem to be harnessing local and cross-context challenges for good, driving the evolution and improvement of CBHPE overall. Below, we synthesize and reflect on the lessons learned from the five most common challenges and adaptations that affect most, if not all, of the core components and that were observed across settings and contexts.

*Faculty and learner development require attention across all core components:* Faculty and learners must be trained in the outcomes framework, which often represents a major change from the objectives-based, structure-process models of traditional educational systems. They also must understand how those outcomes are sequenced to optimize learner development. Additionally, faculty members who direct courses or clinical experiences must understand the importance of tailored learning experiences and competency-focused instruction as the foundation for the learners’ journeys to competence. Finally, all learners and faculty members require ongoing, iterative development to understand and effectively engage with the program of assessment, rather than relying on one-off training interventions. In fact, faculty and learner development in the program of assessment offers a real opportunity to differentiate assessment in CBE from prior programs of assessment in the old structure/process model of education. CBE emphasizes formative assessment for learning, usually through direct observation of daily activities. This focus can improve confidence in summative assessments and entrustment decisions (assessment of learning) as a direct result of increased aggregate data informing decisions and a greater focus on direct observation in CBE.

*Change management is required throughout CBHPE adoption*: From planning to design to implementation to maintenance, leaders in CBHPE need to attend to change management to ensure the program’s uptake and continuous improvement. CBHPE represents a substantial shift in how competence is conceptualized, taught, and assessed [97]. Change management theory consistently emphasizes that such educational transformations can only occur with continuous and iterative cycles of implementation, feedback, and refinement [98]. Frameworks from implementation science and organizational change highlight the importance of context and local adaptation, reflecting on experiences, and continuous quality improvement [99]. In this view, challenges identify misalignment between the intended design and implementation in the real-world, while adaptations address those challenges in context for long-term sustainability.

*Constructive alignment challenges run across CBHPE core components:* Constructive alignment is essentially a *sine qua non* of CBHPE, as it is an outcomes-based approach to developing an educational program [100]. Learners construct meaning through their learning activities (tailored learning experiences and competency-focused instruction). Thus, those learning activities must be aligned with the desired outcomes. Similarly, to ensure the learner has achieved the desired outcomes, the program of assessment must be designed to measure those same outcomes to support both learning and progression decisions. Achieving and sustaining CBHPE requires constant attention to this constructive alignment.

*Time variable, competency-based advancement is a challenge across CBHPE programs:* Most UME and GME programs across the globe are steeped in fixed-time traditions. Accreditation and certification organizations often underscore those traditions with definitive time-in-training requirements. We are aware of only three mature programs with true competency-based time variable progression [101]. The first is from a single residency program in Orthopedics in Toronto [102]. The second was a small pilot project across four institutions referenced above, entitled “Education in Pediatrics Across the Continuum (EPAC)” [31]. The third is from a single residency in Pathology at Massachusetts General Hospital [39,40,41]. These programs have been well described in the literature and often had to fight “uphill battles” to overcome the many barriers to time-variable advancement at the programmatic, system, and national levels.

*Intentional program evaluation is required*: Mature CBHPE programs also uniformly had a robust program evaluation that captured performance in each of the five Core Components, in addition to learner outcomes beyond the competencies (such as standardized test pass rates, program completion rates, match rates in countries with a residency matching program, and quality of care measures). To the extent possible, then, programs in the planning and design stages should build in a clear program of evaluation to examine fidelity to the core components, desired outcomes for the learners and other engaged groups (e.g. faculty and educators), to drive continuous improvement as the CBHPE program goes through progressive iterations. Taking lessons from implementation science, there is a need for evaluation to drive evolution, with subsequent continuous monitoring and adjustments based on measures of the impact of the adaptations made [7].

## Conclusion

In this paper, we describe the key challenges and adaptations that have occurred following the implementation of CBHPE, organized using the Core Components of CBHPE framework [9]. We do not purport to address the scope of challenges; rather, we highlight common challenges experienced and the adaptations that have been implemented successfully in response. In doing so, we highlight how CBHPE is evolving globally. A notable observation across the examples presented is the generic nature of many challenges; that is, they tend to be common across many contexts, countries, program sizes, health professions, and levels of implementation, including local, regional, or national initiatives. Thus, the examples highlighted above were chosen from among numerous potential examples. In the aggregate, these examples underscore both the breadth of challenges associated with sustaining CBHPE and the diversity of contextually responsive adaptations that have emerged internationally. At the same time, however, we recognize that most of our examples come from the medical profession, and a majority of those come from North America. This is a direct result of our focus on adaptations following implementation and, therefore, focused on mature programs. Competency-based medical education had an earlier start than other health professions, by as much as a decade or more, with some of the earliest national efforts in graduate medical education in the US and Canada. Thus, the literature is unavoidably weighted towards the medical profession in North America.

We hope this paper provides those in any phase of their CBHPE program journey with some practical insights into the common challenges and adaptations experienced globally after implementation. By focusing on the evolution of CBHPE programs through responsive adaptations to anticipated and novel challenges, we highlight the dynamic and iterative nature of change. We further hope that by sharing these experiences, our global community of educators can work together and learn from each other to advance CBHPE in preparing healthcare professionals to be better able to meet the increasingly complex needs of the societies we serve.

## AI statement

Non-native English authors relied on AI for translation/language support.

## Disclaimer

The views expressed herein are those of the authors and not necessarily those of the American Medical Association, the ChangeMedEd consortium participant schools, the Association of American Medical Colleges, the American Board of Pediatrics, the American Board of Pediatrics Foundation, The Royal College of Physicians and Surgeons of Canada, or other Federal or Governmental Agencies.
